# Process evaluation of the context, reach and recruitment of participants and delivery of dementia-specific case conferences (WELCOME-IdA) in nursing homes (FallDem): a mixed-methods study

**DOI:** 10.1186/s13063-018-3147-9

**Published:** 2019-01-14

**Authors:** Daniela Holle, Rene Müller-Widmer, Sven Reuther, Ute Rosier-Segschneider, Rabea Graf, Martina Roes, Margareta Halek

**Affiliations:** 1 German Center for Neurodegenerative Diseases (DZNE), Stockumer Str. 12, 58453 Witten, Germany; 2 Faculty of Health, University of Witten/Herdecke, Alfred-Herrhausen-Straße 50, 58455 Witten, Germany

**Keywords:** Dementia-specific case conference, Behavioural and psychological symptoms, Process evaluation, Nursing home, Dementia, Mixed-methods study, WELCOME-IdA

## Abstract

**Background:**

A system of dementia-specific case conferences (WELCOME-IdA) was evaluated using a stepped-wedge design in six nursing homes (NHs) to enable nursing staff to analyse properly the behavioural and psychological symptoms (BPSD) of residents with dementia. A process evaluation of the context, the recruitment and target populations reached (residents and nursing staff) and the delivery of the intervention and implementation strategy was carried out to explore the lack of effectiveness of WELCOME-IdA on the residents’ prevalence of BPSD.

**Methods:**

This study was part of a larger process evaluation using a mixed-methods design. Standardised questionnaires, semi-structured interviews, attendance lists, standardised protocols and written self-reports were used to collect the data. The quantitative data were analysed by calculating descriptive statistics. The qualitative interviews were analysed using deductive content analysis and the self-reports were analysed using a documentary analysis.

**Results:**

None of the NHs had prior experience with dementia-specific case conferences on a specific concept related to BPSD. The number of residents for whom a dementia-specific case conference was held was lower than expected. The number of nursing staff reached was high, although as defined in the study protocol, core nursing teams continuously participating in all components of the intervention were not established throughout the study. An analysis of the delivery of the intervention showed a reduction in the frequency of dementia-specific case conferences and deviations in the process structure and role structure of WELCOME-IdA. The strategy used to facilitate the implementation of WELCOME-IdA was mostly followed. An analysis of the recruitment of residents indicated that the variation in which residents were included in the study sample was high and that the intended sample size required to achieve a power >80% was not reached.

**Conclusion:**

An analysis of the process evaluation data indicated that there were inaccuracies in the implementation of WELCOME-IdA and there were methodological limitations within the design of the effectiveness trial, both of which could explain the lack of effectiveness of WELCOME-IdA. To optimise the process structure of WELCOME-IdA, an assessment of BPSD and a pre-selection of possible domains for the behavioural analysis could be conducted prior to a dementia-specific case conference.

**Trial registration:**

ISRCTN20203855. Registered on 10 July 2013.

## Background

The behavioural and psychological symptoms of dementia (BPSD) affect up to 70% of nursing home (NH) residents with dementia [1]. They are associated with poor outcomes, such as the enhanced use of psychotropic medications [2, 3] and a decreased quality of life [4], and they are predictors of hospitalisation [5] and progression to severe dementia and death [6]. BPSD increase the burden on NH staff [7, 8]. The effectiveness of any treatment for BPSD relies on an accurate identification of the precipitating determinants of the symptoms, which then become the targets of interventions [9].

BPSD result from interactions among a broad range of patient, caregiver and environmental determinants that are idiosyncratic to each person with dementia [10]. In the FallDem study, a system of dementia-specific case conferences (DSCCs) titled *Wittener Modell der Fallbesprechung bei Menschen mit Demenz mit Hilfe des Innovativen-demenzorientierten-Assessmentsystems* (WELCOME-IdA) was implemented in NHs to enable nursing staff to analyse properly the precipitating triggers of BPSD in NH residents [11]. To facilitate the implementation of WELCOME-IdA into routine care, a parallel implementation strategy was introduced.

The effectiveness of WELCOME-IdA was analysed using a stepped-wedged cluster randomised controlled trial in six NHs (clusters) [12]. There was no difference in the overall prevalence of BPSD between the control and intervention phases (primary outcome) [0.09%, 95% confidence interval −0.01; 0.18] [13]. However, based on the results of an earlier study, a reduction in the prevalence of BPSD of 12% from the control to the intervention phase was expected [14]. To explore potential deviations between the expected and observed outcomes in the FallDem trial, a process evaluation was conducted in parallel with the effectiveness study [15]. Process evaluations can provide insight into the so-called black box of effectiveness studies and determine whether failure is attributable to the intervention, poor implementation or methodological limitations due to the design [16]. Process evaluations contribute to the understanding of how and to what extent interventions are delivered in daily practice [17–19]. Furthermore, examining the extent to which an intervention reaches its intended target populations is vital for establishing the extent to which the outcome evaluation represents a valid test of the intervention theory [19]. Knowing the sampling quality in terms of the recruitment of the target populations is also important for drawing conclusions about the external validity of the study results [17, 20, 21]. Finally, process evaluations can provide contextual insight into usual care and the circumstances prior to the implementation of an intervention [15, 19].

For the process evaluation of the FallDem study, the framework suggested by Grant et al. [18] for designing process evaluations of cluster-randomised trials was used. Among other aspects, this framework considers the context, recruitment of individuals, individuals reached and the delivery to clusters as essential domains to be studied in a process evaluation. Therefore, this paper addresses the following five different research questions: (1) What is the context in which the intervention is implemented? (2) How were the participants in the intervention recruited by the cluster and which individuals in the target population (residents and NH staff) actually received the intervention? (3) Was the intervention delivered as intended in each NH (cluster)? (4) Who actually received the implementation? (5) Was the implementation delivered as intended in each NH (cluster)?

## Methods

### Study design

This study was part of a larger process evaluation using a mixed-methods design [15]. The process evaluation was carried out during a stepped-wedge cluster randomised controlled trial into the effects of WELCOME-IdA [12] (ISRCTN20203855). In each cluster, the process data were gathered at baseline (T0) and during the 7-month intervention phase.

### Intervention

**Fig. 1:**
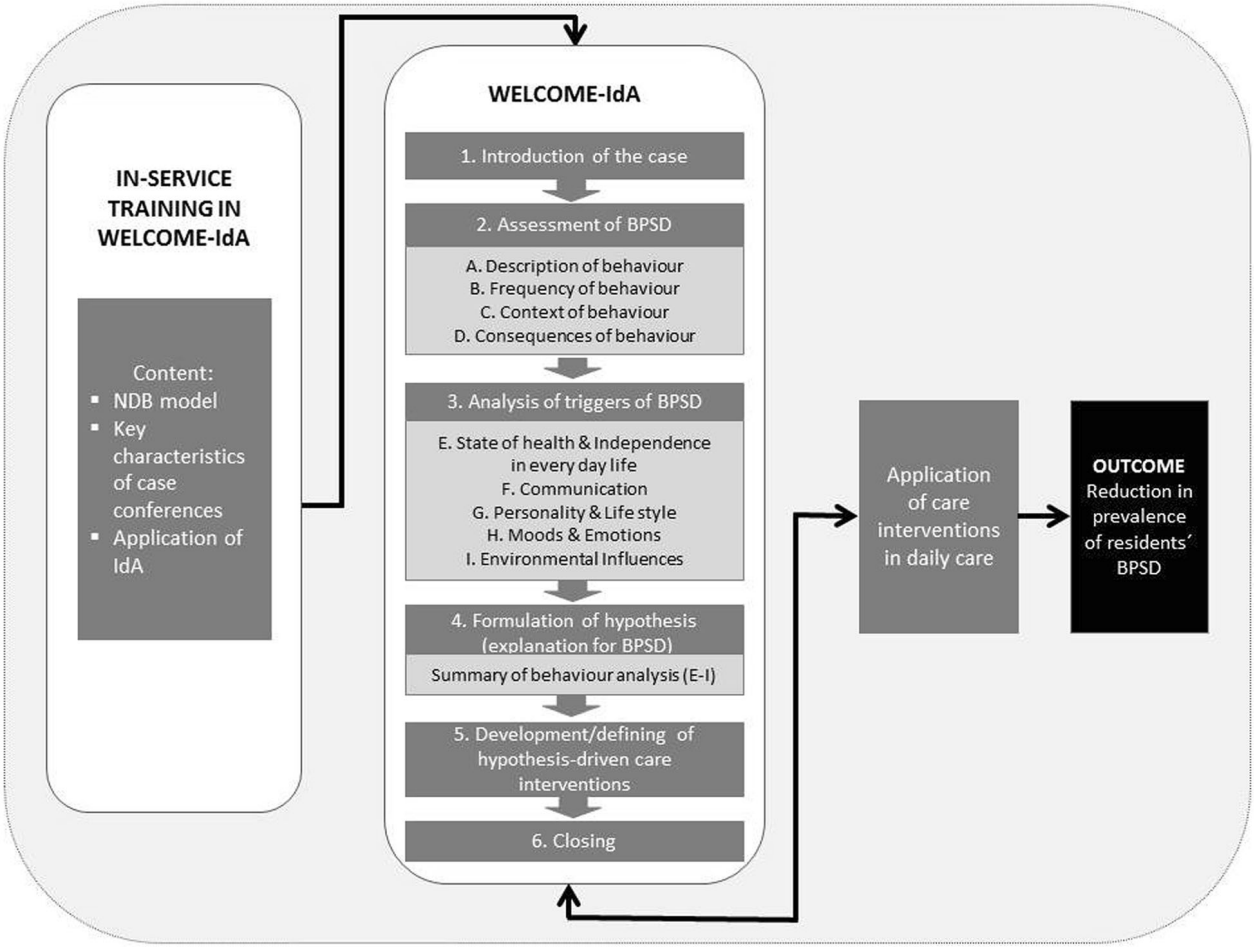
Model for the implementation of the system of dementia-specific case conferences WELCOME-IdA. BPSD behavioural and psychological symptoms of dementia, NDB need-driven compromised behaviour

The DSCCs in WELCOME-IdA form a structured, goal-directed, intra-professional procedure that supports nursing staff in the assessment and analysis of the triggers of BPSD in residents [11]. WELCOME-IdA is embedded in the general theory of hermeneutics and the need-driven compromised behaviour model [22, 23]. In general, hermeneutics describes the philosophy of understanding and interpreting the social interactions among individuals, groups and organisations [24]. Moreover, hermeneutics can be used to interpret observed behaviour and it strengthens the ability of nursing teams to understand the perspectives of people with dementia with respect to their social or biographical background [22, 25]. The need-driven compromised behaviour model applies a more specific theory that provides explanations for the triggers underlying BPSD exhibited by individuals with dementia. Thus, the aim of WELCOME-IdA is to identify and analyse the triggers that commonly cause BPSD in people with dementia. Based on behavioural analysis, a hypothesis-driven care intervention was planned and introduced into nursing practice to address the triggers of BPSD and, thus, reduce or prevent BPSD among residents (Fig. 1).

WELCOME-IdA has a fixed process structure. Each case conference has six steps:

1. introduction
2. assessment of BPSD
3. analysis of the triggers of BPSD
4. formulation of a hypothesis
5. development of hypothesis-driven care interventions
6. close

Steps 2–4 are supported by a comprehensive assessment system called the Innovative dementia-oriented Assessment System (IdA) [26]. IdA provides four domains with guiding questions to assess BPSD (A: a description of the behaviour, B: the frequency of the behaviour, C: the context of the behaviour and D: the consequences of the behaviour) and five domains with guiding questions to analyse the triggers of BPSD (E: state of health and independence in everyday life, F: communication, G: personality and life style, H: moods and emotions and I: environmental influences). The assessment ends with a summary of the analysis of the identified triggers of BPSD (E-I), which supports the formulation of a hypothesis. In addition to the process structure, WELCOME-IdA includes a predefined role structure (a moderator, a case reporter, a keeper of minutes and 2 to 5 reflection partners). Both, the process and role structure should support systemising the reflection of the case and prevents the nursing staff from digressing in everyday conversations or developing hasty conclusions and action plans. A WELCOME-IdA conference can last between 60 and 90 min and should be conducted at least once a month, preferably in a room without distractions.

Due to the stepped-wedge design of the effectiveness study, the intervention was integrated in practice in one NH every 3 months over a period of 19 months. The final step included two NHs instead of one because one NH functioned as an optional replacement if the other NH dropped out of the study. Each intervention phase began with in-service-training on WELCOME-IdA, followed by four on-the-job training sessions on WELCOME-IdA per team (months 1–3). Then, a minimum of four DSCCs were performed without any assistance (WELCOME-IdA off-the-job training phase) by the nursing teams (months 4–7) (Table 1).

**Table 1:** Overview of the implementation of the intervention using a stepped-wedge design

No. of nursing homes	Month					
	1–3	4–6	7–9	10–12	13–15	16–19
1	I	I	F	F	F	F
1	C	I	I	F	F	F
1	C	C	I	I	F	F
1	C	C	C	I	I	F
2	C	C	C	C	I	I

*C* control phase, *I* intervention phase, *F* follow-up phase

### Parallel implementation strategy

To facilitate the integration of the intervention in practice, an implementation strategy (parallel to the effectiveness study) was initiated. First, a kick-off meeting with senior management, quality management and head ward nurse representatives discussed the key elements of WELCOME-IdA. Following the meeting with these representatives, a second kick-off meeting was organised at each NH to provide information about WELCOME-IdA to the participating nursing teams (wards). Additionally, a steering group was established at each NH. The steering group was responsible for the implementation process (such as providing structural requirements and the designation of responsibilities). This group was also responsible for conducting an assessment (at the beginning of the study) of the strengths and weaknesses of their organisation in relation to the context in which the case conferences would be conducted. Based on the results of this assessment, a tailored implementation plan was developed for each NH. Therefore, the steering group received three coaching sessions by an external trainer. Finally, additional in-service training sessions comprising the modules ‘Dementia and BPSD’ and ‘Moderator Skills’ were offered (Table 2). The implementation strategy was based on the results of a previous feasibility study [27] and the general understanding that to achieve the sustainability of the implementation of WELCOME-IdA in practice requires communication through all existing organisational channels. The full content of WELCOME-IdA has been outlined elsewhere [12].

**Table 2:** Overview of time schedule, participants and workload of the components of the intervention and implementation strategy in one NH

When	Week	Components of intervention and implementation strategy	Participants	Workload (total of 64.5 h)
4 weeks before the start of the intervention		Kick-off meeting with senior management	Representatives of senior management, quality management and head nurses of nursing teams	3 h
		Kick-off meeting with nursing staff	Nursing staff of nursing teams	1.5 h
		1st coaching session with steering group	Senior management and head nurses of nursing teams	3 h
Weeks 1–10 (3 months)	1	In-service training on ‘Dementia and BPSD’	All staff including nursing teams	3 h
	2	In-service training on WELCOME-IdA, 1st day	Managers of nursing teams, head nurses, nursing teams and moderators	6 h (3 h per team)
		In-service training on ‘Moderation skills’, 1st day	2 people from each nursing team for a total of 4 people	6 h
	3, 4	In-service training on WELCOME-IdA, 2nd day	Managers of nursing teams, head nurses, nursing teams and moderators	6 h (3 h per team)
		In-service training on ‘Moderation skills’, 2nd day	2 people from each nursing team for a total of 4 people	6 h
	5	1st on-the-job training session on WELCOME-IdA	Nursing teams and moderator	6 h (3 h per team)
	6	2nd on-the-job training session on WELCOME-IdA	Nursing teams and moderator	6 h (3 h per team)
	7	3rd on-the-job training session on WELCOME-IdA	Nursing teams and moderator	6 h (3 h per team)
	8	2nd coaching session of steering group	Senior management and head nurses of nursing teams	3 h
	9	4th on-the-job training session on WELCOME-IdA	Nursing teams and moderator	6 h (3 h per team)
	10	3rd coaching session of steering group	Senior management and head nurses of nursing teams	3 h
Weeks 13–28 (4 months)	13–16	1st WELCOME-IdA off-the-job-training phase	Nursing teams and moderator	60–90 min
	17–20	2nd WELCOME-IdA off-the-job-training phase	Nursing teams and moderator	60–90 min
	21–24	3rd WELCOME-IdA off-the-job-training phase	Nursing teams and moderator	60–90 min
	25–28	4th WELCOME-IdA off-the-job-training phase	Nursing teams and moderator	60–90 min

*BPSD* behavioural and psychological symptoms of dementia, *NH* nursing home

### Data collection

#### Context

A self-developed standardised questionnaire was used to assess the organisational characteristics (size, number of units, characteristics of the residents and employees, sick leave and advanced training of the nursing staff) of the 6 participating clusters and 12 participating nursing units. The nursing managers of the six NHs completed the questionnaire at the baseline. The items on the questionnaire were considered appropriate based on a prior quasi-experimental study [28]_._

Semi-structured telephone interviews [29] were conducted with a head ward nurse at each participating nursing unit at baseline to explore whether the nursing teams already had experience of case conferences and, in the teams with prior experience, to determine how they ran the case conferences (frequency, duration, role structure, participants and process structure). Two dementia researchers conducted the interviews. Each interview was audiotaped and transcribed verbatim.

#### Recruitment and target populations reached

To evaluate the sampling quality, the recruitment and informed consent procedures were standardized across NHs and documented by the research team. The attendance lists and standardised protocols of each DSCC were used to assess which NH residents and NH nursing staff were reached during the 7-month intervention phase. The attendance lists and standardised protocols were previously pilot tested in one NH and determined to be appropriate.

#### Delivery of intervention and implementation strategy

The attendance lists and standardised protocols were also used to assess the delivery of the intervention and the implementation strategy. Standardised protocols were used to document each DSCC that occurred during the 7-month intervention phase and provide information on the adherence to the process structure, the role structure and other key characteristics (frequency, duration and location). The trainers completed the attendance lists and protocols during the on-the-job training phase, and the participating nursing teams during the DSCC completed them during the off-the-job training phase.

Nonconformities in the in-service training, on-the-job training or coaching of the training curricula were documented by self-reports (written documentation) by the trainers and coaches after the training.

### Data analysis

The quantitative data were analysed with IBM SPSS Statistics version 21 by calculating descriptive statistics (frequencies, percentages, means and standard deviations). All qualitative interviews were analysed using deductive content analysis [30]. Based on the semi-structured interview guide, a structured analysis matrix was developed. Two researchers coded the interviews to improve the inter-subjectivity and comprehensibility of the results. Deviations between the interview codes were discussed by the research team, and a consensus was reached. The self-reports of the trainers were analysed using documentary analysis [31], which means that differences between the plan for the sessions and the actual coaching and trainings sessions were analysed. The reference for the analysis was the written curriculum for the coaching and training sessions. All analyses were regularly presented to and discussed by the research team.

## Results

### Context

#### Structural characteristics of the NHs and units

Three of the NHs were non-profit organisations, one NH was a public organisation and two NHs were for-profit organisations. The NHs had a mean size of 82.2 residents (54–100; Table 3), which is above the national average of 63 residents [32]. Two units were selected from each NH. Most residents had low to moderate levels of care dependency according to an assessment of their long-term care needs, which primarily comprises functional abilities. The levels of care dependency are consistent with the average national distribution of care levels in NHs [32]. On average, 51.8 people worked as care staff in the six NHs. Cluster E82 had the fewest nursing staff (*n* = 38). Half of the nursing staff were registered nurses (which requires 3 years of vocational training), which complies with the legal regulations for German NHs [33]. NHs E89 and E50 hired the most new nursing staff (≥4 employees) in the last 3 months.

**Table 3:** Structural characteristics of the NHs (units)

Feature	Nursing home											
NHs enrolled at baseline	E29		E79		E89		E75		E82		E50	
Nursing home size (*n*)	79		100		80		80		54		100	
Number of units (*n*)	3		4		2		2		2		2	
Residents’ level of care dependency [%]												
0	3.8		0		2.5		0		0		1.0	
1 (low)	29.1		36.0		32.5		42.5		42.6		35.0	
2 (moderate)	35.4		25.0		47.5		30.0		42.6		40.0	
3 (severe)	29.1		33.0		15.0		25.0		9.3		20.0	
3+ (very severe)	2.5		6.0		2.5		1.3		0		1.0	
Number of total nursing staff (*n*)	51		59		53		60		38		50	
Number of registered nurses* (*n*)	29		30		21		21		17		23	
New employees, last 3 months	2		1		4		1		2		5	
Employees resigned, last 3 months	1		1		1		0		MD		3	
Units enrolled at baseline	Unit 1	Unit 2	Unit 1	Unit 2	Unit 1	Unit 2	Unit 1	Unit 2	Unit 1	Unit 2	Unit 1	Unit 2
Unit size (*n*)	26	26	26	26	41	39	40	40	30	24	49	51
Residents´level of care [%]												
0	0	0	0	0	2.4	2.6	0	0	0	0	0	0
1 (mild)	34.6	26.9	26.9	42.3	34.1	25.6	37.5	47.5	46.7	37.5	34.7	37.3
2 (moderate)	30.8	42.3	19.2	34.6	51.2	46.2	25.0	35.0	40.0	45.8	38.8	43.1
3 (severe)	26.9	30.8	42.3	23.1	7.3	23.1	37.5	12.5	13.3	4.2	24.5	15.7
3+ (very severe)	7.7	0	11.5	0	2.4	2.6	0	2.5	0	0	0	2.0
Total nursing staff (*n*)	17	18	20	13	18	23	25	26	20	18	25	27
Number of registered nurses (*n*)	10	9	7	8	7	9	8	8	9	8	11	11
Sick leave of the total nursing staff [hrs], last 3 months	297	495	341	386	347.6	509.4	458	358.6	328	318	1080	1410
Advanced training of the total nursing staff [hrs], last 3 months	124	182	71	46	88.6	5	456.3	526	40	40	46.5	34

*Number is independent of the number of hours worked by each nurse

*MD* missing data, *NH* nursing home

At the unit level, the average size of the units was 35 residents (24–51). In four clusters (E89, E75, E82 and E50), all residents were reached, since they had two units each. As at the cluster level, most residents were mildly to moderately dependent on care. The average number of nursing staff per unit was 20.8 (13–27). The average of the total number hours of sick leave taken by the nursing staff in a unit during the last 3 months was 433 (297–1410). Cluster E50 had the highest number of hours of sick leave (1080 and 1410). The average of the total number of hours of advanced training for all nursing staff in a NH was 138.3 (5–456.3).

#### Usual care before the study

Nine of the 12 nursing teams (units) had experience with case conferences prior to the implementation of WELCOME-IdA, although these case conferences were neither restricted to nor relied on a specific concept related to BPSD among people with dementia.

The frequency of the case conferences varied within the teams. Some occurred weekly (E29), monthly (E50), quarterly (E79 and E75) or irregularly based on demand (E89). The duration of the case conferences also varied within the teams. The ranges were as follows: 0.25–0.5 h (E89), 0.5–1 h (E50); 1 h (E29, E79 and E75) and 2 h (E29). Most case conferences were held during lunchtimes (E29, E79 and E89). The moderator of the case conferences was mainly a manager (head ward nurse or deputy or care manager; E29, E89, E50 and E75). Usually the primary caregiver presented the case (E29, E79 and E75). In three clusters (E29, E79 and E89), the case reporter was also the moderator (dual role). In another team (E79), the case reporter was additionally responsible for taking the minutes of the case conferences (triple role). The case conferences were recorded in writing in four of the nine teams (E29, E89 and E75).

Depending on the NH, the participants of the case conferences varied. In all clusters, the nursing staff on duty on the day of the case conferences and the nursing staff working in the resident’s nursing ward participated in the case conferences (E29, E79, E89, E75 and E50). In one cluster, two additional people from a different residential area who did not know the case (resident) also participated in the case conferences (E75). Depending on the case, most clusters also invited social services staff (E29, E79, E89, E75 and E50) and occasionally invited relatives, a physician, psychologists, kitchen staff or managers of the NHs (E79, E89 and E50).

All clusters had guidelines for their case conferences. The guidelines ranged from a 12-page assessment tool (E29) addressing different topics (e.g., eating/drinking, allergies, bedsores, falling, and pain contractures) that were then systematically reviewed and discussed in the case conference to standardised protocols with overarching guiding questions (e.g., What is the problem? What are the goals? What are the resources? What are the interventions?) (E50). The main intention of the case conference was to assess and update the nursing plan (E89 and E29). Thus, in one team, the nursing plan was read aloud during the case conference (E89).

### Recruitment and individuals reached: intervention

#### Recruitment of units and nursing staff

In each NH, the management selected two units (nursing teams) for participation in the study according to the inclusion criteria [12]. In all NHs, the two units recruited at least 30 residents with dementia for the study. The care of a resident predominantly occurred in their home unit. For the effectiveness study, each participating unit was required to establish one core nursing team (5–8 people, including 2 moderators) to participate in all parts of the intervention, since the components of the intervention built upon each other and the intervention requires continuous learning.

#### Nursing staff reached in the intervention

On average, 13.8 people (5–22) participated in the first in-service training session on WELCOME-IdA, and 14.6 people (6–25) participated in the second in-service training session on WELCOME-IdA. The participants represented a skill mix of registered nurses and nursing assistants, and the number of registered nurses clearly predominated in all NHs. Social service staff members were present in E29 and E89, and senior management were present in E79, E89 and E75. Almost all people selected to be trained as moderators of the DSCCs also attended the in-service training session on WELCOME-IdA (Table 3).

WELCOME-IdA recommends that core nursing teams of 5–8 people participate in each case conference, which was partially observed during the WELCOME-IdA on-the-job training and WELCOME-IdA off-the-job training phases. In two clusters (E29 and E79), the group size was at least twice as large, whereas in cluster E82, the group size was mostly not reached (<5 people). The participants represented a mix of registered nurses and nursing assistants, and the number of registered nurses clearly predominated in all NHs. The leading ward nurses were almost always present in clusters E29 and E75 but almost always absent in cluster E82. Social service staff members were routinely present in E29 but only occasionally in clusters E79, E89, E75 and E82.

Comparing the absolute number of participants (*N*) with those who participated continuously (core team) in the intervention, only cluster E29 realised the continuous participation of at least five core members. The people who continuously participated in all components of the intervention were mostly the skilled moderators (Table 4).

**Table 4:** Reach of nursing staff in components of the intervention

Phase	Nursing home											
	E29		E79		E89		E75		E82		E50	
	*N* [MOD]		*N* [MOD]		*N* [MOD]		*N* [MOD]		*N* [MOD]		*N* [MOD]	
1st in-service training in WELCOME-IdA	19 [6]		22 [8]		15 [3]		9 [5]		5 [3]		n/a	
2nd In-service training in WELCOME-IdA	17 [6]		25 [9]		16 [5]		11 [5]		6 [4]		n/a	
	Unit 1	Unit 2	Unit 1	Unit 2	Unit 1	Unit 2	Unit 1	Unit 2	Unit 1	Unit 2	Unit 1	Unit 2
	*N* [MOD]	*N* [MOD]	*N* [MOD]	*N* [MOD]	*N* [MOD]	*N* [MOD]	*N* [MOD]	*N* [MOD]	*N* [MOD]		*N* [MOD]	*N* [MOD]
1st WELCOME-IdA on-the-job training	8 [5]	11 [6]	14[4]	MD	6 [2]	5 [2]	9 [5]	8 [4]	4 [3]		n/a	n/a
2nd WELCOME-IdA on-the-job training	10 [6]	12 [6]	16 [6]	MD	n/a	10 [4]	6 [3]	9 [4]	6 [1]		n/a	n/a
3rd WELCOME-IdA on-the-job training	11 [6]	11 [6]	9 [5]	MD	–	7 [4]	7 [4]	7 [4]	4 [1]		n/a	n/a
4th WELCOME-IdA on-the-job training	9 [6]	10 [6]	17 [4]	8 [1]	n/a	n/a	6 [4]	6 [4]	4 [1]		n/a	n/a
1st WELCOME-IdA off-the-job training	15 [5]	12 [5]	n/a	n/a	n/a	n/a	MD	n/a	n/a		n/a	n/a
2nd WELCOME-IdA off-the-job training	15 [4]	11 [5]	10 [3]	7 [3]	n/a	n/a	n/a	MD	n/a		n/a	n/a
3rd WELCOME-IdA off-the-job training	19 [6]	17 [6]	10 [4]	8 [2]	n/a	n/a	n/a	MD	MD		n/a	n/a
4th WELCOME-IdA off-the-job training	14 [5]	15 [5]	12 [2]	7 [1]	n/a	n/a	MD	n/a	n/a		n/a	n/a

*MD* missing data, *MOD* number of skilled moderators

#### Recruitment and residents reached for data collection

The inclusion and exclusion criteria [12] were provided to the NH management of the participating clusters for the initial selection of NH residents eligible for the study. Based on a previous power calculation [12], each cluster should recruit 30 residents with dementia for the primary outcome (prevalence of BPSD) to achieve a total sample size of at least *n* = 150 residents. During the study, deceased residents (dropouts) were replaced by newly admitted residents. In total, 57 residents participated in all data measurements, 87 residents dropped out and 57 residents were newly admitted after the baseline. The newly enrolled residents had lower levels of care dependency, were less cognitively impaired, reported less pain and showed less BPSD than the residents who were enrolled at baseline.

**Fig. 2:**
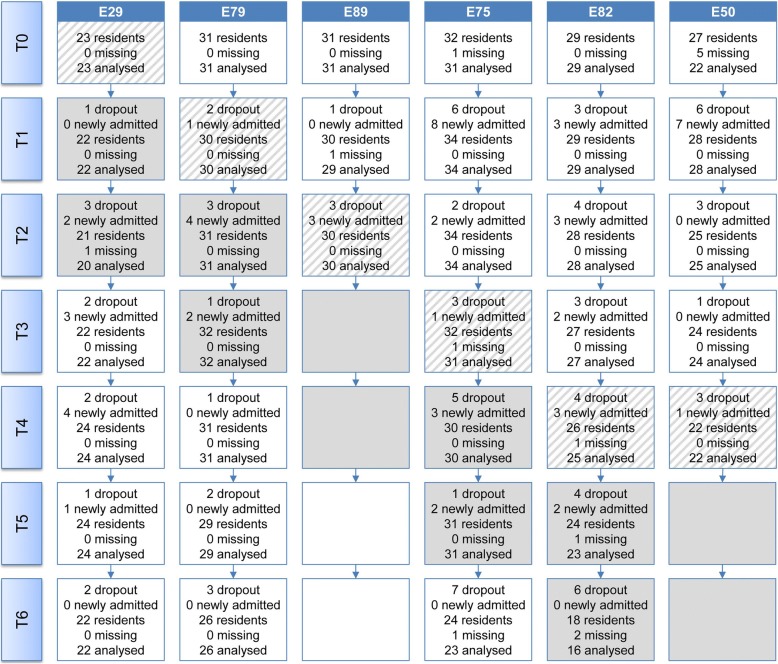
Results of the recruitment of residents in the FallDem study

The residents’ participation rate varied per cluster and per measurement (T0–T6; Fig. 2). All clusters continuously contributed to the residents’ sample, except for the two for-profit organisations (E89 and E50), which dropped out of the study after T2 and T4, respectively. Thus, the required sample size of *n* = 150 was no longer reached after the third data collection time point (T3).

`

#### Residents reached in the intervention

Based on the results of an earlier study [14], seven residents (cases) in each nursing ward should have directly received the intervention and could be discussed in a DSCC (*n* = 3 during the on-the-job training on WELCOME-IdA and *n* = 4 during the WELCOME-IdA off-the-job training phase). This results in a total of 84 cases if all cases from the 12 participating teams are combined. It was assumed that the knowledge acquired about the cases discussed would be applied to other residents involved in the study (radiation effect in terms of the residents’ primary outcome). Furthermore, the nursing teams where trained to select residents for WELCOME-IdA who were also included in the data collection (primary outcome).

In total, *n* = 33 cases were discussed during the on-the-job training on WELCOME-IdA, including 23 residents for whom data were also collected. Two of the 23 residents were discussed twice. In total, 87% of these residents showed at least one challenging behaviour (CB) in the data collection prior to the case conference. The mean of the total index (frequency × severity) for the Neuropsychiatric Inventory (Nursing Home Version) (NPI-NH) for the 23 residents was 12, 7 (standard deviation 11, 4). During the WELCOME-IdA off-the-job training phase, 20 residents were discussed, including 14 residents who were included in the data collection. Four of the 14 residents were previously discussed during the on-the-job training on WELCOME-IdA. Of these residents, all showed at least one CB in the data collection prior to the case conference and the mean NPI-NH total index for them was 13, 8 (standard deviation 11, 8). Overall, the mean NPI-NH total index of the residents discussed in the case conferences was compatible with the mean NPI-NH total index of the total resident sample at baseline.

### Delivery of intervention

#### Frequency and duration of the intervention

While implementing WELCOME-IdA, 75% (18/24) of the proposed in-service and 66.7% (32/48) of the on-the-job training sessions on WELCOME-IdA occurred during the 3-month on-the-job training phase. In one NH (E82), one instead of two teams (units) joined the training due to limited resources. In one team (E29, unit 1), the first on-the-job training session was used to clarify the remaining organisational questions. Two teams in E89 ended the on-the-job training prematurely, and E50 did not participate in any training (Table 4).

Following the training, the clusters performed 39.6% (19/48) of the proposed DSCCs during the off-the-job training phase. The decrease in WELCOME-IdA can be mainly explained by the dropout of two clusters (E89 and E50) and because one cluster started the intervention with only one instead of two teams (E82) (Table 4).

The training on WELCOME-IdA and the on-the-job training on WELCOME-IdA each lasted 3 hours according to the curriculum, except for the final training session in E82, which was abridged (2.25 h). All WELCOME-IdA conferences in the off-the-job training phase lasted between 60 and 90 min. One NH (E29) decided to expand the duration of each DSCC to 2 hours.

#### Process structure of WELCOME-IdA

The process structure of WELCOME-IdA was maintained in 78.1% (25/32) of WELCOME-IdA on-the-job and 78.9% (15/19) of the WELCOME-IdA off-the-job training sessions. No information is available for 3 (32) WELCOME-IdA on-the-job and 1 (19) WELCOME-IdA off-the-job training sessions due to missing data.

The deviations from the process structure were characterised because twice no care planning (phase 5) was performed during an on-the-job training session due to the lack of time (E29). Consequently, the participating NH (E29) changed the process structure of WELCOME-IdA in the third on-the-job training session. Subsequently, the assessment of the behaviour (phase 2) of the resident being discussed in the DSCC was performed by the case reporter prior to the DSCC. The case reporter also selected single domains of IdA for the behaviour analyses (phase 3) and completed the IdA questionnaire prior to the DSCC. During the DSCCs, the case reporter provided a short summary of the results of the behaviour assessment and the behaviour analyses. The nursing teams in the DSCC discussed these results. This change in the methodology of IdA was adopted by the external trainers in E79 and E82 to give participants more time to understand and reflect on the results of the assessment.

Despite this change in applying WELCOME-IdA, no care interventions were planned during two additional DSCCs in the off-the-job training phase, and once, the assessment tool was not used because a topic other than BPSD was discussed (E82).

#### Role structure in case conferences

The role structure of WELCOME-IdA was maintained in 59.4% (19/32) of the WELCOME-IdA on-the-job and 47.4% (9/19) of the WELCOME-IdA off-the-job training sessions, although 9.4% (3/32) of the data sets were missing during the on-the-job training sessions and 26.3% (5/19) of the data sets were missing during the off-the-job training sessions. Deviations from the role structure were characterised by the fact that fewer reflection partners (<2) were present during WELCOME-IdA (E82). Skilled moderators and a case reporter were present during all on-the-job and off-the-job training sessions.

#### Location of the intervention

All in-service training, on-the-job training and off-the-job training sessions on WELCOME-IdA were performed in a room free of distractions.

### Participants reached: implementation

The average number of participants in the in-service training on ‘Dementia and BPSD’ was 22.4 (15–27), including a mixture of registered nurses (*n* = 50), nursing assistants (*n* = 28), social services staff (*n* = 10) and other individuals (*n* = 24).

The in-service training sessions on ‘Moderation Skills’ were attended by an average of 5.8 (4–9) people. The participants were exclusively registered nurses or people with a leadership role (e.g., a leading ward nurse or head of social services). In cluster E29, only people without management functions were deliberately selected for the training on moderation skills (E29). Hence, except for cluster E29, at least two moderators participated in the coaching of the steering group. Cluster E29 decided to include a moderator in the steering group after the first coaching session of the steering group to facilitate communication between the moderators and the steering group (E29). On average, 4.9 (2–7) people participated in the coaching of the steering group. Generally, the senior management and two leading ward nurses of the participating nurses constituted the steering group. In some NHs (E79, E89 and E82), the head of social services or a representative of the quality management team (E75) was also present during the coaching of the steering group.

### Delivery of implementation strategy

All kick-off meetings (*n* = 12) were realised as planned in the six NHs. All in-service training sessions on dementia, BPSD and moderation skills were delivered as planned, except in cluster E50. All coaching sessions of the steering groups were performed, except for clusters E89 and E50. The second and third coaching sessions of the steering groups were abridged (second coaching session: mean 2.71 h, third coaching: mean 2.53 h). The trainers reported no other deviations in the components of the implementation strategy from the curriculum.

## Discussion

The aim of this paper was to describe the process of applying and integrating the system of DSCCs in WELCOME-IdA in six NHs as well as to explore the deviations between the observed and expected outcomes. The framework suggested by Grant et al. [18] for designing process evaluations of cluster-randomised trials was used in the FallDem trial [15].

### Context

The six NHs varied in structural and organisational characteristics, which obviously had an impact on the application and integration of the intervention into daily care routines. While the smallest NH (E82) with the fewest nursing staff members per head decided to unite both units at the beginning of the on-the-job training on WELCOME-IdA to allocate resources, in E29 and E79, an above-average number of people participated in all components of the intervention, although continuous participation by the same staff members did not occur. An explanation for the high participation rate in E29 and E79 might be that the staff members in these units who did not participate in the trial joined the intervention to benefit from the training. Consequently, the moderators had to manage large groups with various levels of knowledge about the DSCC, which contrasts with the intention of the intervention to train core nursing teams [12]. Both changes may have diminished the effectiveness of WELCOME-IdA as fewer residents were reached, and the degree of penetration of the intervention may have been lowered because continuous learning of cases was possible only to a limited extent due to the lack of continuity of participants.

Moreover, the nursing staff in all NHs took high levels of sick leave over the 3 months prior to baseline, indicating that the remaining nursing staff in the NHs were subjected to a high workload and time pressure. These conditions definitively hinder the application and integration of new innovations such as WELCOME-IdA and might explain the reduced number of DSCCs at the end of the intervention phase and the premature dropout of two NHs, both of which also impacted on the effectiveness of WELCOME-IdA. High workloads and time pressure were also mentioned as the most widely reported barriers to applying and integrating case conferences in routine care in NHs in other studies [27, 34].

The analysis of usual care indicated that most NH teams performed general case conferences prior to the implementation of WELCOME-IdA, which is consistent with previous research investigating case conferences in German NHs [35, 36]. The variation in how NHs perform case conferences highlights that there are currently no national or international standards for key elements of case conferences [34]. To implement WELCOME-IdA successfully, considerable re-organisation is needed in NHs, as the intervention cannot be directly integrated into existing routine structures.

In line with this study, in future research, a local analysis of the structural and organisational characteristics of the NHs as a collaboration by both researchers and users of the intervention should be performed prior to the implementation of the intervention to determine whether NHs have the resources to implement a new intervention and determine how to attune the implementation strategy to the local situation. Participatory action research could be a first step to make the implementation a joint effort by both researchers and users of the intervention [37, 38].

### Recruitment and target populations reached

Only 57 of the resident sample participated in all measurements (T0–T6) and the sample size of 150 residents required to achieve a power >80% was not realized due to the loss of two NHs. A contributing factor to the loss of two clusters might be that the NHs and study participants, including the nursing staff, did not receive any financial incentives or gifts, both of which are promising retention strategies for in-person follow-ups in health-care studies [39]. Although a combination of different non-financial retention strategies were used in the FallDem study (e.g. a letter of intent jointly signed by the managers of the NHs, the research team and the educational centre who delivered the WELCOME-IdA training; the appointment of a study coordinator at each NH; a systematic and tailored method for contacting and scheduling appointments with the study coordinators; telephone reminders; a detailed study description of the requirements, potential benefits and risks; the creation of a study identity using a logo and similar colours and fonts on all study materials), this did not prevent two NHs from dropping out. Against the backdrop of limited personnel resources in NHs, future third-party funding for NH research should consider financial support and reimbursements for NHs to facilitate research activities and thus, optimise the retention of study participants.

The number of target residents reached in the intervention was low, since some residents were discussed twice in different case conferences, the nursing staff selected residents who were not included in the study sample and the number of DSCCs was reduced. These aspects might explain the lack of effectiveness of WELCOME-IdA on the prevalence of BPSD. The Norwegian TIME study investigating the effects of DSCC on agitation in NH residents achieved 91% of the required residents (*n* = 104), and DSCCs were found to be effective [40].

Furthermore, in this study, the nursing teams were responsible for deciding which residents they would like to discuss in the DSCCs [12]. This decision can be very different depending on the subjective opinion of the teams. In this study, residents with a mean NPI-NH total index of 12.7 during the on-the-job training phase and a mean NPI-NH total index of 13.8 in the off-the-job training phase were discussed in the DSCCs. These values are low compared to the range of the NPI-NH total index of 0–144, where a higher score indicates more behavioural disturbances [41]. An alternative approach would have been to determine a priori via the NPI-NH index which residents should be included in the sample and thus, also addressed in the case conferences. In the study by Lichtwarck et al. [40], a moderate to high degree of agitation (defined as NPI subscale index ≥6) was a prerequisite for the selection of study participants. The freedom of choice for the selection of case residents might have had an impact on the effectiveness of WELCOME-IdA. In future studies, the criteria for selecting cases for DSCCs should be carefully considered. The evaluation of problematic behaviour cannot be derived solely from the nursing staff’s subjective assessment of the frequency and severity of a resident’s behaviour. Other aspects, such as the subjective burden of the person with dementia, the context and the competence of the nursing staff in addressing the behaviour, should also be taken into account [42, 43].

As previously outlined, the analysis of the participation lists showed that many staff members were reached through the intervention; however, continuous participation by the same staff during the different components of the intervention, which build upon each other, did not occur. The lack of continuity might have decelerated the learning processes of the individual nursing staff members, especially those performing the roles of case reporter and reflection partner, and thus, prolonged the time needed to produce a change in the behaviour of nursing staff regarding how to manage BPSD and to produce a change in the prevalence of BPSD among NH residents. Thus, the radiation effects emanating from a trained nursing team may hardly have been expected to occur. Moreover, the lack of core nursing teams cannot be compensated for by the continuity of moderators. Moderators are crucial for goal-oriented communication during a DSCC, but their task is not to introduce the case, reflect on the case and finally transfer the results of the DSCC into all nursing practices [12].

Nevertheless, the moderators became key people who participated in the coaching of the steering groups to facilitate communication among all groups involved in the implementation of WELCOME-IdA. The participation of the moderators in the coaching of the steering group was not foreseen in the conception of the parallel implementation strategy but seems to be useful for future implementation.

### Delivery of intervention and implementation strategy

Due to (1) the dropping out of two NHs, (2) the decision of E82 to combine two nursing teams and (3) the decision of E75 to perform WELCOME-IdA without monthly support in the rotation between the two nursing teams, the frequency of WELCOME-IdA was significantly reduced, which may have contributed to the lack of effectiveness of the intervention [13]. The duration of all components of the intervention and most components of the parallel implementation strategy were consistently adhered to, thus providing no indication of why the intervention had no effect on BPSD. The duration of the coaching sessions was abridged, which may be because nearly all the NHs had prior experience in performing case conferences and that likely not all topics needed to be discussed in detail in the coaching of the steering groups. The deviations in the process structure of WELCOME-IdA show that in some cases, no care interventions were planned during a DSCC to address BPSD, and on one occasion, BPSD was not the topic of the DSCC. Both occurrences might have also had an impact on the success of WELCOME-IdA, as the introduction of care interventions is essential for producing change in BPSD. The performance of IdA in the beginning of the application of WELCOME-IdA might be a reason for the lack of intervention planning during the case conferences, which mostly occurred in E29. In the beginning, IdA was used exclusively in case conferences, suggesting that nursing teams spent considerable time answering the questions in IdA. Consequently, during the first on-the-job training, it was decided with the trainers that the case reporter should use IdA in the preparation of the case. Performing IdA before the case conference allows for more time to be spent on understanding and reflecting on the results of the assessment and developing hypothesis-driven interventions related to the behaviour analysis. In future DSCCs, the approach might be to use an assessment of the case already prepared to assess the BPSD and preselect topics provided by IdA to analyse the behaviour as previously proposed by other approaches related to the behaviour analysis of NH residents [44, 45].

Deviations in the number of reflection partners were observed only in the smallest NH, which might indicate that it is even more challenging for a smaller NH to integrate DSCCs into routine care if a group size of at least five people is required. Performing DSCCs in a room free of distractions emerged as an easily realisable goal, whereas in a previous study, the absence of a quiet room was a barrier hampering the performance of DSCCs in NHs [27].

### Limitations

The in-service WELCOME-IdA training, the delivery of WELCOME-IdA as well as the application of hypothesis-driven care interventions into daily care interact with each other and are central elements impacting the outcome of the FallDem trial. Although the delivery of the in-service training in WELCOME-IdA and the performance of WELCOME-IdA were closely monitored within this process evaluation, no data were systematically collected to provide insight into the treatment fidelity of the care interventions. Future research should assess the application of the care interventions in daily care, for example, using direct observations or written care plans.

## Conclusions

The process evaluation of the context, recruitment, the target population reached, delivery of the intervention and parallel implementation strategy in the FallDem trial indicates that the lack of effectiveness of WELCOME-IdA might partially be explained by implementation error. Implementation error can be characterised by the following: (1) the low number of residents for whom a DSCC was held, (2) a reduction in the frequency of DSCCs, (3) a lack of personal continuity among nursing staff who participate in the different components of the intervention and (4) hindrances in the continuous learning. Moreover, in some NHs, care interventions were not planned during a DSCC or IdA was not used because the topic addressed was not related to BPSD, which might have further contributed to the lack of significant effectiveness of WELCOME-IdA.

Methodological challenges in the effectiveness trial and stepped-wedge designs, such as the variation in which residents were included in the sample and that the intended power of >80% was not reached in the FallDem trial, might further explain the absence of a significant effect of WELCOME-IdA.

The process evaluation data also provided information on how to optimise the process structure of WELCOME-IdA. In future DSCCs, the approach might be to use the IdA assessment already prepared for the case to assess BPSD and preselect topics provided by IdA to analyse the behaviour of a resident.

The moderators functioned as key personnel and facilitated the implementation of WELCOME-IdA. In future implementations, it would seem expedient that moderators also participate in the coaching of the steering group to facilitate communication among all groups involved in the implementation of WELCOME-IdA.

Further analyses of the response of clusters and the attitudes of nursing staff regarding the key elements of WELCOME-IdA are needed to draw final conclusions about its application and integration into daily care routines as well as the effectiveness of WELCOME-IdA on residents’ prevalence of BPSD.

## Abbreviations

BPSD: Behavioural and psychological symptoms of dementia
CC: Case conference
DSCC: Dementia-specific case conference
IdA: Innovative-demenzorientierte Assessmentsystem
MD: Missing data
MOD: Number of skilled moderators
NDB: Need-driven compromised behaviour
NH: Nursing home
NPI-NH: Neuropsychiatric Inventory (Nursing Home Version)
WELCOME-IdA: Wittener Modell der Fallbesprechung bei Menschen mit Demenz mit Hilfe des Innovativen-demenzorientierten Assessmentsystems
